# RIS-Assisted Cooperative Time-Division Multiple Access

**DOI:** 10.3390/s24010178

**Published:** 2023-12-28

**Authors:** Hyukmin Son, Beom Kwon

**Affiliations:** 1 Department of Electronic Engineering, Gachon University, Seongnam 13120, Republic of Korea; hson102@gachon.ac.kr; 2 Division of Interdisciplinary Studies in Cultural Intelligence, Dongduk Women’s University, Seoul 02784, Republic of Korea

**Keywords:** beamforming, MIMO, RIS, TDMA

## Abstract

Reconfigurable intelligent surface-aided communication systems have been intensively investigated to improve capacity, coverage, and energy efficiency via optimal controlling of phase shifts for passive reflecting elements. However, there are few studies on cooperative transmission incorporating RIS in TDMA systems, because RIS reflects all the incident signals, and it inadvertently leads to a boost in interference signals. In this paper, we propose RIS-assisted cooperative time-division multiple access, in which the required SINR of all users is guaranteed as much as possible by opportunistic use of RIS for cooperative transmission. The proposed scheme’s primary function is that some time slots, i.e., cooperative time slots, serve a pair of users, i.e., a strong- and a weak-channel-conditioned user, using RIS. To support this functionality, we propose scheduling for non-cooperative and cooperative time slots, user pairing for scheduled cooperative time slots, and transmit beamforming vector design for the pair of UEs in each cooperative time slot. The simulation and numerical results demonstrate that the proposed scheme guarantees QoS for all UE as much as possible and minimizes the remaining required capacity indicating the amount of capacity that was not achieved compared with the required capacity.

## 1. Introduction

With the evolution of wireless communications toward 5G-advanced, terrestrial wireless communications have integrated other communications such as satellite communications, IoT networks, and symbiotic ambient backscatter communications for high spectral efficiency and ubiquitous access to massive devices in high-density areas [1,2,3]. Together with integrated communication systems, reconfigurable intelligent surfaces (RISs) have recently been recognized as a promising technology for future 6G communications [4,5]. RISs integrate a large number of passive reflecting elements with low hardware cost and energy consumption [6,7,8]. With digital reconfigurable and programmable metasurface technology, RISs can adeptly modify the amplitude and phase of the incident signal and reflect it constructively, improving spectrum efficiency and coverage capability [9,10,11].

Due to their significant advantages, RIS-aided communication systems have been intensively investigated with numerous related research endeavors. The authors in [9,10] jointly optimized the transmit beamforming at the base station (BS) and passive beamforming at the RIS to minimize the total transmit power at BS in multiple-input single-output (MISO) downlink systems. This study showed that a RIS-aided MIMO system achieved the same performance as a massive benchmark MIMO system with significantly reduced active antennas/RF chains. In [9], the authors focused on continuous phase shifts at reflecting elements, while in [10], they focused on discrete phase shifts at reflecting elements considering practical implementation. In [12], the authors demonstrated that RIS can establish a virtual line-of-sight (LoS) between BS and user equipment (UE), resulting in enhanced coverage and signal-to-noise ratio (SNR) in mmWave systems. In the study presented in [13], the authors devised energy-efficient designs that encompass both the allocation of transmit power and the determination of phase shifts for the passive reflecting elements. These designs were developed while adhering to individual link budget guarantees for the users. The author in [14] optimized precoding at BS and phase shift values at RIS to balance the trade-off between spectral efficiency and energy efficiency. The works mentioned above addressed intricate phase optimization challenges that pose computational intensity and real-time implementation difficulties. Crucially, these algorithms relied on precise channel state information (CSI) for the link from BS to UE across the RIS. There is much research on channel estimation in RIS-assisted systems, but it is worth noting that channel estimation still has many difficulties and issues for practical implementation, such as its accuracy, computational complexity, and overhead [15,16,17,18,19,20].

The random phase shift-based opportunistic scheduling can be utilized as an intriguing alternative approach to overcome the abovementioned issues in RIS-assisted communications [21,22,23,24,25,26]. In other words, BS dynamically serves the UE experiencing the most favorable instantaneous channel condition at any given moment under the given random phase shift on the RIS, not focusing on optimizing the phase shift on the RIS. In [24], the authors analyzed the sum capacity by incorporating the fading effects of both direct links (i.e., between BS and UE) and reflection links (i.e., between BS, RIS, and UE). This capacity was expressed as a function of both the number of users and the number of meta-atoms. The authors in [25] demonstrated that the throughput of the RIS-assisted opportunistic communication system asymptotically converged to the optimal beamforming-based throughput when the number of users was large. In [26], opportunistic scheduling was used to increase energy-efficient performance while turning on/off using multiple RISs spatially distributed to serve wireless users. The authors focused on the joint optimization problem of transmit beamforming and RIS control, aiming to maximize the energy efficiency under minimum rate constraints of the users. However, the RIS-assisted opportunistic communication to serve two UEs in the same time slot can still have a unique challenge because RISs can inadvertently amplify interference signals between each other. Since time slot-based resource allocation methods are used in most communication systems, including LTE and NR, studying RIS-based opportunistic transmission methods in TDMA systems has a valuable contribution from the perspective of developing a technique that can be applied to many commercialized communication systems. From the perspective of practicality, guaranteeing the quality-of-service (QoS) of scheduled UEs is more important than maximizing the sum capacity of scheduled UEs.

In this paper, we propose RIS-assisted cooperative time-division multiple access (RC-TDMA), in which the required signal-to-interference-plus-noise ratio (SINR) of all users is guaranteed as much as possible by opportunistic use of RIS for cooperative transmission. In the proposed RC-TDMA, each user has a time slot dedicated to each user. The time slots are divided into two groups: non-cooperative time slots and cooperative time slots. If any scheduled users in the time slots are expected to be satisfied with QoS, it is called a strong-channel-conditioned UE; these time slots become the candidate time slots to perform cooperative transmission using the RIS. Conversely, suppose any scheduled users in the time slots are not satisfied with QoS, then it is called a weak-channel-conditioned UE. In that case, these users become candidates for additional data service in the cooperative time slots. Thus, a pair of users, i.e., strong- and weak-channel-conditioned UEs, can be simultaneously served in the time slot dedicated to the strong-channel-conditioned UE. Thus, the RIS is only turned on and operated in the cooperative time slot for the cooperative transmission. We adopt a random phase shift-based RIS, which is constant for the given time slots. It allows us to estimate the effective channel between BS and UE without computational complexity or overhead. Therefore, in this study, we propose scheduling for non-cooperative and cooperative time slots, user pairing (i.e., weak-channel-conditioned UE and strong-channel-conditioned UE) for a scheduled cooperative time slot, and transmit beamforming vector design for both UEs in the cooperative time slots. The simulation and numerical results demonstrate that the proposed RC-TDMA ensures QoS for all UEs as much as possible. Thus, the proposed scheme minimizes the remaining required capacity indicating the amount of capacity that was not achieved compared with the required capacity. Furthermore, it is shown that the proposed scheme can increase power efficiency compared with the conventional TDMA that does not use the RIS when the RIS consumes much less power than BS power consumption.

### Notations

For the notations, we use uppercase boldface for matrices and lowercase boldface for vectors. Symbols $A^{H}$ indicates the conjugate transpose of matrix A. Similarly, $A^{T}$ and $A^{-1}$ indicate the transpose and inverse (or pseudoinverse) of matrix A, respectively. Symbol |a| denotes the absolute value of a complex number *a*, and ∥a∥ indicates $\ell_{2}$-norms of vector a.

## 2. RIS-Assisted Cooperative Time-Division Multiple Access

### 2.1. System Model

**Figure 1:**
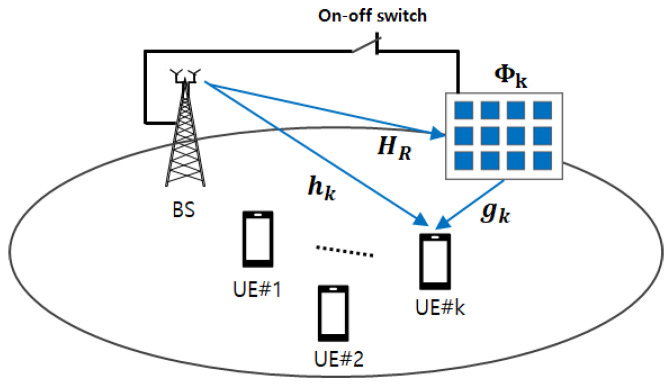
System model for RIS-assisted cooperative time-division multiple access.

**Figure 2:**
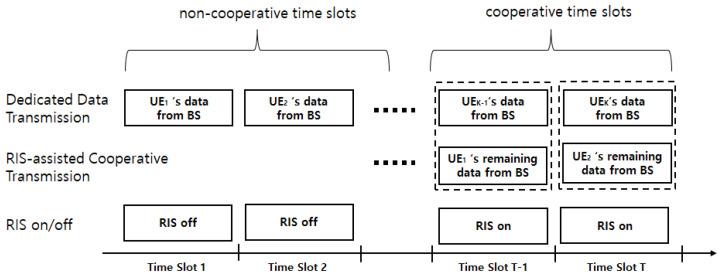
An example of time slot allocation for RIS-assisted cooperative time-division multiple access.

We consider a multiuser downlink TDMA, which consists of *K* UEs, one RIS, and one BS, as shown in Figure 1. Each UE, RIS, and BS has a single antenna, *N* passive reflecting elements, and *M* transmit antennas, respectively. A frequency flat block fading channel is assumed among them so that each channel is constant within a block. The total number of time slots in a block is *T*, the total number of UEs in a block is *K*, and each time slot is allocated to each UE, as shown in Figure 2. Since each UE has a dedicated time slot, the total number of UEs is the same as the total number of time slots, i.e., T=K.

In conventional TDMA systems, each UE is served once in the dedicated time slot. However, in the proposed TDMA system, the UE, which does not meet the required SINR, can be served once more, i.e., two UEs can simultaneously be served in a time slot dedicated to the UE that satisfies the required SINR. When serving two UEs in the time slot, the RIS turns on and then assists the data transmission of the UE that does not meet the required SINR. The proposed system’s primary goal is that all users’ required SINR is guaranteed as much as possible by opportunistic use of the RIS for cooperative transmission.

Estimation methods for wireless channels with RISs are being actively studied [15,16,17,18,19,20]. However, it is difficult to estimate the channels between BS-RIS and RIS-UE separately. Thus, a randomly given phase shift on the RIS is used in a block, which enables a UE to estimate the effective channel for BS-RIS-UE. Since each UE can estimate the effective channel from BS and feed it back to BS, it can be assumed that BS has perfect CSI for the effective channel from BS to each UE.

In the proposed RC-TDMA system shown in Figure 1, the BS schedules UEs in increasing order of channel gain so that the earlier time slots are allocated to the UEs with a weak channel condition. Inversely, the later time slots are allocated to UEs with a strong channel condition. If some UEs allocated to the earlier time slots cannot satisfy the required capacity, they can have an opportunity to receive the remaining data at the later time slots. In other words, their data can be transmitted at the later slot with the strong-channel-conditioned UEs’ data. Figure 2 shows an example of time slot allocation for the RC-TDMA.

In this example, $UE_{1}$ and $UE_{2}$ are the weak-channel-conditioned UEs. Thus, BS allocates them to the front time slot. Since the required capacities of UEs cannot be guaranteed, time slot 1 and time slot 2 are solely dedicated to $UE_{1}$ and $UE_{2}$, respectively. Subsequently, in the case of $UE_{K-1}$ and $UE_{K}$, if their required capacities can be sufficiently guaranteed, BS performs user pairing between the strong-channel-conditioned UE (i.e., $UE_{K-1}$ or $UE_{K}$) and the weak-channel-conditioned UE (i.e., $UE_{1}$ or $UE_{2}$) and serves two scheduled UEs at the same time for guaranteeing the required capacity of the weak-channel-conditioned UE as much as possible. These slots are called cooperative time slots. The other time slots, except the cooperative time slots, are called non-cooperative time slots. At this time, time slots K−1 and *K* are dedicated slots for $UE_{K-1}$ and $UE_{K}$, respectively. Thus, the data transmission for $UE_{1}$ and $UE_{2}$ must not interfere with $UE_{K-1}$ and $UE_{K}$. In the non-cooperative time slots, RIS maintains a turn-off for power efficiency. In the cooperative time slots, the RIS turns on and performs a passive relay to maximize the capacities of two UEs. Hereafter, the indexes of both UE and time slot are omitted for simplicity.

In a non-cooperative time slot, the received signal of the UE is given as

(1) $$y_{n}=\sqrt{P}hw_{n}x+n,$$

where *P* is the total transmit power, *x* denotes the transmit symbol of the UE, $E\left[{\left|x\right|}^{2}\right]=1$, $h(\in C^{1\times M})$ is the channel vector from BS to the UE, $w_{n}\left(\in C^{M\times 1}\right)$ is the transmit beamforming vector for transmitting *x*, and *n* is the additive white Gaussian noise with zero mean and variance $N_{0}$. The received SINR and capacity in the non-cooperative time slot are then given as

(2) $$\begin{array}{c} {\mathrm{SINR}}_{n}=\rho {∥hw_{n}∥}^{2}, \end{array}$$

(3) $$\begin{array}{c} C_{n}=log_{2}\left(1+{\mathrm{SINR}}_{n}\right), \end{array}$$

where ρ is the transmit SNR, i.e., $\rho =\frac{P}{N_{0}}$.

In a cooperative time slot, the received signal of the weak-channel-conditioned UE is given as

(4) $$y_{c,w}=\underset{desired\;signal\;term}{\underbrace{\sqrt{P_{w}}g_{w}\Phi Hw_{c,w}x_{w}+\sqrt{P_{w}}h_{w}w_{c,w}x_{w}}}+\underset{interference\;signal\;term}{\underbrace{\sqrt{P_{s}}g_{w}\Phi Hw_{c,s}x_{s}+\sqrt{P_{s}}h_{w}w_{c,s}x_{s}}}+n,$$

where $\sqrt{P_{w}}$ and $\sqrt{P_{s}}$ are, respectively, the allocated transmit power to the weak-channel-conditioned and strong-channel-conditioned UEs, $x_{s}$ denotes the transmit symbol of the strong-channel-conditioned UE, $E\left[|x_{s}{|}^{2}\right]=1$, $g_{w}\left(\in C^{1\times N}\right)$ is the channel vector from RIS to the weak-channel-conditioned UE, Φ is a N×N random phase-shift diagonal matrix of the RIS, $w_{c,w}\left(\in C^{M\times 1}\right)$ is the transmit beamforming vector for transmitting $x_{w}$ in the cooperative time slot, and $w_{c,s}\left(\in C^{M\times 1}\right)$ is the transmit beamforming vector for transmitting $x_{s}$ in the cooperative time slot. The received SINR of the weak-channel-conditioned UE in the cooperative time slot can be expressed as

(5) $${\mathrm{SINR}}_{c,w}=\frac{\rho_{w}{∥\left(g_{w}\Phi H+h_{w}\right)w_{c,w}∥}^{2}}{1+\rho_{s}{∥\left(g_{w}\Phi H+h_{w}\right)w_{c,s}∥}^{2}},$$

where $\rho_{w}$ and $\rho_{s}$ are, respectively, the transmit SNRs of the weak-channel-conditioned and strong-channel-conditioned UEs in the cooperative time slot, i.e., $\rho_{w}=\frac{P_{w}}{N_{0}}$, $\rho_{s}=\frac{P_{s}}{N_{0}}$, and $\rho =\rho_{w}+\rho_{s}$.

In a cooperative time slot, the received signal of the strong-channel-conditioned UE is given as

(6) $$\begin{array}{ccc} y_{c,s} & = & \underset{desired\;signal\;term}{\underbrace{\sqrt{P_{s}}g_{s}\Phi Hw_{c,s}x_{s}+\sqrt{P_{s}}h_{s}w_{c,s}x_{s}}}+\underset{interference\;signal\;term}{\underbrace{\sqrt{P_{w}}g_{s}\Phi Hw_{c,w}x_{w}+\sqrt{P_{w}}h_{s}w_{c,w}x_{w}}}+n, \end{array}$$

(7) $$\begin{array}{ccc} & = & \sqrt{P_{s}}g_{s}\Phi Hw_{c,s}x_{s}+\sqrt{P_{s}}h_{s}w_{c,s}x_{s}, \end{array}$$

where $g_{s}\left(\in C^{1\times N}\right)$ is the channel vector from the RIS to the strong-channel-conditioned UE and $h_{s}\left(\in C^{1\times M}\right)$ is the channel vector from BS to the strong-channel-conditioned UE. As mentioned above, in the cooperative slots, data transmission for the weak-channel-conditioned UE must not interfere with the strong-channel-conditioned UE. Thus, the beamforming vector of $w_{c,w}$ should be designed to satisfy $\left(g_{s}\Phi H+h_{s}\right)w_{c,w}=0$. Based on $w_{c,w}$ nullifying the interference signal term in Equation (6), Equation (7) is derived. The received SINR of the strong-channel-conditioned UE in the cooperative time slot can be then expressed as

(8) $${\mathrm{SINR}}_{c,s}=\rho_{s}{∥\left(g_{s}\Phi H+h_{s}\right)w_{c,s}∥}^{2}.$$

### 2.2. Beamforming Vector Design

In this section, for a given random phase shift matrix of RIS, Φ, we determine and derive optimal transmit weight vectors, $w_{n}$, $w_{c,s}$, and $w_{c,w}$ to maximize ${\mathrm{SINR}}_{n}$, ${\mathrm{SINR}}_{c,s}$ and ${\mathrm{SINR}}_{c,w}$, respectively. Based on these analyses, the RC-TDMA algorithm is proposed to guarantee the required capacity of the weak-channel-conditioned UE as much as possible.

To maximize the capacity of a weak-channel-conditioned UE in a non-cooperative time slot, i.e., Equation (3), the maximum ratio transmission (MRT) beamforming is adopted, which is given as

(9) $$w_{n}=\frac{h^{H}}{∥h∥}.$$

For a cooperative time slot, the transmit beamforming vector for the strong-channel-conditioned UE should be determined to maximize the SINR of Equation (8), which can be rewritten as

(10) $$\max_{w_{c,s}}{\mathrm{SINR}}_{c,s}=\max_{w_{c,s}}\rho_{s}w_{c,s}^{H}{\left(g_{s}\Phi H+h_{s}\right)}^{H}\left(g_{s}\Phi H+h_{s}\right)w_{c,s}\;\;\;\;s.t.\;\;{∥w_{c,s}∥}^{2}=1.$$

By applying the eigenvalue decomposition to Equation (10), the optimal $w_{c,s}$ for maximizing ${\mathrm{SINR}}_{c,s}$ is obtained by

(11) $$w_{c,s}^{*}=\max.\;\mathrm{eigenvector}\;\mathrm{of}\;{\left(g_{s}\Phi H+h_{s}\right)}^{H}\left(g_{s}\Phi H+h_{s}\right).$$

Note that the optimal $w_{c,s}$ is determined regardless of the value of $\rho_{s}$ in Equation (11). Based on the optimal $w_{c,s}$, the minimum transmit SNR of $\rho_{s}$ to satisfy the required SINR can be calculated as

(12) $$\rho_{s}^{*}=\frac{\gamma_{req}}{{\left(w_{c,s}^{*}\right)}^{H}{\left(g_{s}\Phi H+h_{s}\right)}^{H}\left(g_{s}\Phi H+h_{s}\right)w_{c,s}^{*}},$$

where $\gamma_{req}$ is the required SINR. The required capacity for each UE can then be given as $C_{req}=\log_{2}\left(1+\gamma_{req}\right)$. The reason for using the minimum $\rho_{s}$ to meet the required SINR is to maximize the SINR of Equation (5) by allocating the transmit power to the weak-channel-conditioned UE as much as possible. Thus, the transmit SNR for the weak-channel-conditioned UE is given as

(13) $$\rho_{w}^{*}=\rho -\rho_{s}^{*}.$$

In a cooperative time slot, the transmit beamforming vector for the weak-channel-conditioned UE should be designed to maximize the SINR of Equation (5) while guaranteeing $\left(g_{s}\Phi H+h_{s}\right)w_{c,w}=0$. Thus, we first design the transmit beamforming vector that maximizes the SINR of Equation (5), and the optimal transmit beamforming vector $w_{c,w}^{*}$ is then determined by projecting it onto the null space of $g_{s}\Phi H+h_{s}$ so that $w_{c,w}^{*}$ can satisfy $\left(g_{s}\Phi H+h_{s}\right)w_{c,w}^{*}=0$.

By applying $w_{c,s}^{*}$ determined in Equation (11), the SINR of Equation (5) can be rewritten as

(14) $${\mathrm{SINR}}_{c,w}=\frac{\rho_{w}w_{c,w}^{H}\left(H^{H}\Phi^{H}g_{w}^{H}g_{w}\Phi H+h_{w}^{H}h_{w}\right)w_{c,w}}{1+\rho_{s}{∥\left(g_{w}\Phi H+h_{w}\right)w_{c,s}^{*}∥}^{2}}.$$

Note that $\frac{\rho_{w}}{1+\rho_{s}∥g_{w}\Phi Hw_{c,s}^{*}{∥}^{2}+\rho_{s}{∥h_{w}w_{c,s}^{*}∥}^{2}}$ is the determined value. Thus, by applying the eigenvalue decomposition to Equation (14), $w_{c,w}^{max}$ for maximizing ${\mathrm{SINR}}_{c,w}$ is obtained by

(15) $$w_{c,w}^{max}=\max.\;\mathrm{eigenvector}\;\mathrm{of}\;\left(H^{H}\Phi^{H}g_{w}^{H}g_{w}\Phi H+h_{w}^{H}h_{w}\right).$$

The null space of $g_{s}\Phi H+h_{s}$, $V_{null}$, can be obtained by the eigenvectors of ${\left(g_{s}\Phi H+h_{s}\right)}^{H}\left(g_{s}\Phi H+h_{s}\right)$, which is given as

(16) $${\left(g_{s}\Phi H+h_{s}\right)}^{H}\left(g_{s}\Phi H+h_{s}\right)=V\Lambda V^{H},$$

where $V=[v_{1},\ldots ,v_{\alpha },V_{null}]$, $v_{i}\in C^{M\times 1}$, $V_{null}\in C^{M\times (M-\alpha )}$, α is the rank of ${\left(g_{s}\Phi H+h_{s}\right)}^{H}\left(g_{s}\Phi H+h_{s}\right)$, and Λ is the diagonal matrix whose diagonal elements are the decreasing ordered eigenvalues. In other words, $V_{null}$ is the space consisting of eigenvectors corresponding to eigenvalue zero. Therefore, the optimal transmit beamforming vector for the weak-channel-conditioned UE, $w_{c,w}^{*}$, is given as

(17) $$w_{c,w}^{*}=\frac{V_{null}V_{null}^{H}w_{c,w}^{max}}{∥V_{null}V_{null}^{H}w_{c,w}^{max}∥}.$$

### 2.3. Proposed Algorithm

Table 1 shows the pseudocode for the RC-TDMA algorithm. In Step 1, the related variables are initialized, in which *T* is the total number of time slots, *K* is the total number of UEs, and $Count_{1}$ and $Count_{2}$ are used for counting the number of weak channel-conditioned and strong conditioned UEs, respectively. Note that $T=Count_{1}+Count_{2}$. $W_{weak}$ and $W_{strong}$ are the matrix to save the transmit beamforming vectors for the weak-channel-conditioned and strong-channel-conditioned UEs, respectively, where each column index of each matrix indicates the time slot index. T is a set of the indexes of time slots selected as cooperative time slots in Step 2. $H_{sort}$ is the channel matrix consisting of the channel vector from BS to each UE, where each vector is sorted in increasing order based on each channel gain. $G_{sort}$ is the channel matrix consisting of the channel vector from RIS to each UE, where each vector is sorted corresponding to $H_{sort}$. Note that the time slots are assigned sequentially to UEs according to the increasing order of each UE’s channel gain. Thus, each UE has a dedicated time slot, and the index of UE is the same as the index of the dedicated time slot.

From lines 5 to 14, the number of weak-channel-conditioned UEs, $Count_{1}$, and the number of strong-channel-conditioned UEs, $Count_{2}$, are calculated based on the comparison between the required SINR (i.e.,$\gamma_{req}$) and the SINR calculated using Equation (2). When calculating $Count_{1}$ and $Count_{2}$, the transmit beamforming vectors are determined using Equation (9) under the assumption that each UE is served in a non-cooperative time slot. After the cooperative time slots are determined in Step 2, the transmit beamforming vectors associated with the cooperative time slots are updated. In line (15), $N_{c}$ is the number of cooperative time slots, which can be determined as a minimum value of either $Count_{1}$ or $Count_{2}$.

In Step 2, the user pairing is performed, which is the user scheduling for a cooperative time slot. If a user pairing is performed in a cooperative time slot, two users, i.e., one weak-channel-conditioned UE and one strong-channel-conditioned UE, are simultaneously served in that cooperative time slot.

In lines 16 to 29, the indexes *k* and *t* corresponding to for-loops indicate the $N_{c}$ weak-channel-conditioned UEs and $Count_{2}$ strong-channel-conditioned UEs, respectively. Note that the first time slot is allocated to the weakest-channel-conditioned UE, and the last time slot is allocated to the strongest-channel-conditioned UE, performed by line 3. When performing the user pairing for a given weak-channel-conditioned UE, the optimal strong-channel-conditioned UE is determined to maximize the SINR of line 24. In other words, the time slot assigned to the optimal strong-channel-conditioned UE becomes the cooperative time slot, and the paired weak-channel-conditioned UE and strong-channel-conditioned UE are served simultaneously in this cooperative time slot. The transmit beamforming vectors used in the cooperative time slot are updated in lines 25 to 28. Consequently, the optimal transmit beamforming vectors are determined, as shown in line 30.

### 2.4. Discussion of System Performance

The objective of the proposed algorithm is that each UE meets the required capacity. In other words, the proposed algorithm ensures QoS, i.e., required SINR or capacity, for all UE as much as possible, not focusing on maximizing the sum capacity for all UEs. Thus, we define the remaining required capacity to measure the performance of the proposed algorithm, indicating the amount of capacity that was not achieved compared with the required capacity.

When the *k*th UE is served only in the allocated non-cooperative time slot (i.e., the dedicated time slot), its remaining required capacity is given as

(18) $$C_{short,RIS}^{k}={\left[C_{req}-C_{n}\right]}^{+},$$

where ${\left[v\right]}^{+}=\max\left(0,v\right)$. When the *k*th UE is served in the allocated non-cooperative time slot, and it is also served in the allocated cooperative time slot as the weak-channel-conditioned UE, its remaining required capacity is given as

(19) $$C_{short,RIS}^{k}={\left[C_{req}-C_{n}-C_{c,w}\right]}^{+}.$$

when the *k*th UE is served in the allocated cooperative time slot as the strong-channel-conditioned UE, its remaining required capacity is given as

(20) $$C_{short,RIS}^{k}={\left[C_{req}-C_{c,s}\right]}^{+}=0,$$

because the proposed algorithm performs power allocation to maximize the weak-channel-conditioned UE’s SINR in the cooperative time slot, and it leads to the decrease in the strong-channel-conditioned UE’s SINR to become the same value as the required SINR, i.e., $C_{req}=C_{c,s}$. Therefore, the total remaining required capacity can be given as

(21) $$C_{short,RIS}=\sum_{k=1}^{K}C_{short}^{k}.$$

In the conventional TDMA system, the remaining required capacity for *k*th UE can be defined as

(22) $$C_{short,BS}^{k}={\left[C_{req}-C_{k}\right]}^{+},$$

where $C_{k}$ is *k*th UE’s capacity in each time slot under the assumption that MRT beamforming is used. Then, the total remaining required capacity in the conventional TDMA system can be given as

(23) $$C_{short,BS}=\sum_{k=1}^{K}C_{short,BS}^{k}.$$

In the proposed algorithm, RIS is turned on to reflect the incident signals only in cooperative time slots. In the cooperative time slots, transmit beamforming vectors at BS are designed to maximize UEs’ SINRs, considering the reflecting effect on the RIS. The on/off mechanism in the proposed algorithm leads to an increase in power efficiency compared with systems that do not use RISs. For example, for the conventional TDMA systems that do not use RISs, additional data should be transmitted using additional time slots, which can provide additional data transmission to users who do not meet the required capacity. Thus, this causes power consumption at the BS. Since the RIS, which delivers signals using passive reflecting elements, is more power-efficient than BS, the proposed algorithm can show better power efficiency than the conventional TDMA. Thus, we can define the effective capacity from the power-efficiency perspective, using the power consumption ratio between BS and RIS. In the conventional TDMA system, the effective capacity can be represented as

(24) $$C_{eff,BS}=\frac{\sum_{k=1}^{K}\min\left(C_{k},C_{req}\right)}{KP_{c}},$$

where $P_{c}$ is the power consumption of BS in each time slot. In the proposed algorithm, the effective capacity can be represented as

(25) $$C_{eff,RIS}=\frac{KC_{req}-C_{short}}{KP_{c}+\tau P_{c}N_{c}},$$

where τ is the power consumption ratio between BS and RIS, i.e., 0<τ<1, and $N_{c}$ is the number of cooperative time slots. In Equations (24) and (25), the effective capacities can be interpreted as measuring satisfaction with the required capacity, i.e., QoS, compared with the power consumption.

## 3. Simulation Results

In this section, simulation and numerical results are provided to validate the performance of the proposed algorithm compared with the conventional TDMA system. The proposed scheme can be used with the other schemes or used alone in the TDMA system. Therefore, in this simulation section, we measure the feasibility of whether the proposed scheme can be combined with the TDMA systems from the perspective of various performance metrics. It is assumed that the channel from BS to RIS, H, is the Rician fading channel, and the channels from the RIS to UE and from BS to UE, i.e., h and g, are Rayleigh fading channels. The main simulation parameters are given in Table 2. For the performance comparison, we adopt the conventional TDMA system, in which each UE is served using MRT beamforming in each dedicated time slot without a RIS.

**Figure 3:**
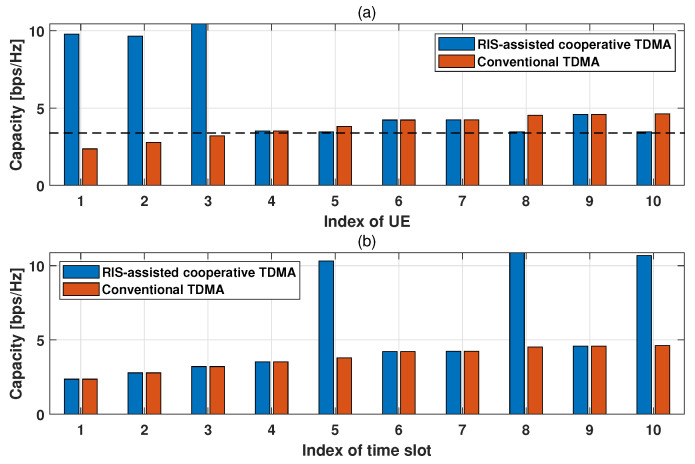
Capacity comparison: (**a**) capacity per UE (**b**) capacity per time slot.

Figure 3 shows the capacity for each UE and time slot under an instantaneous channel when the transmit SNR is 5 dB, i.e., ρ = 5 dB. The dotted line in Figure 3a is the required capacity, $C_{req}$, and time slots are sequentially allocated to UEs that are sorted in increasing order of channel gain, ${∥h∥}^{2}$. For example, time slot 1 is allocated to the weakest-channel-conditioned UE, and time slot 10 is allocated to the strongest-channel-conditioned UE in both TDMA systems. As shown in Figure 3a, in the conventional TDMA, three UEs, i.e., the indexes of UE, 1, 2, and 3, are unsatisfied with the required capacity. In the proposed RC-TDMA, these UEs’ capacities are significantly improved and are satisfied with the required capacity. This is because the weak-channel-conditioned UEs are additionally served in cooperative time slots with the strong-channel-conditioned UEs. As shown in Figure 3b, the capacities of time slots 5, 8, and 10 in the RC-TDMA are significantly higher than the others because these slots are cooperative time slots. In other words, a pair of UEs, i.e., the weak- and strong-channel-conditioned UEs, are served simultaneously in the cooperative time slots. On the contrary, in the conventional TDMA, the capacity of each UE is the same as the capacity of each time slot because there is no additional cooperative transmission.

**Figure 4:**
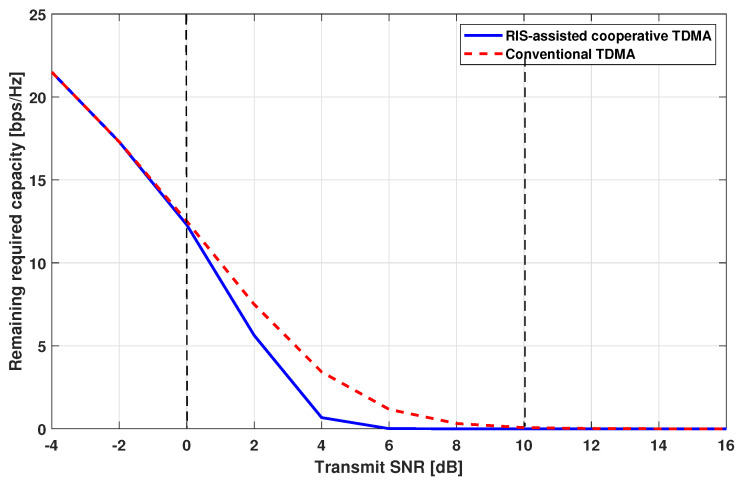
Remaining required capacity comparison.

Figure 4 shows the remaining required capacities, i.e., $C_{short,RIS}$ and $C_{short,BS}$, according to the transmit SNR. As mentioned in Section 2.3, the number of cooperative time slots is determined as a minimum value of either the number of weak-channel-conditioned UEs or the number of strong-channel-conditioned UEs. In the low SNR region, i.e., between −4 dB and 0 dB, both TDMA systems show a similar remaining required capacity because most UEs are weak-channel-conditioned. Conversely, In the high SNR region, i.e., between 10 dB and 16 dB, both TDMA systems show a similar remaining required capacity because most UEs are strong-channel-conditioned. In both cases, there are few opportunities to set the cooperative slots in the RC-TDMA. In the moderate SNR region, i.e., the range between 0 dB and 10 dB, the RC-TDMA shows lower remaining required capacity compared with the conventional TDMA due to the opportunities to perform RIS-assisted transmission in the cooperative time slots.

**Figure 5:**
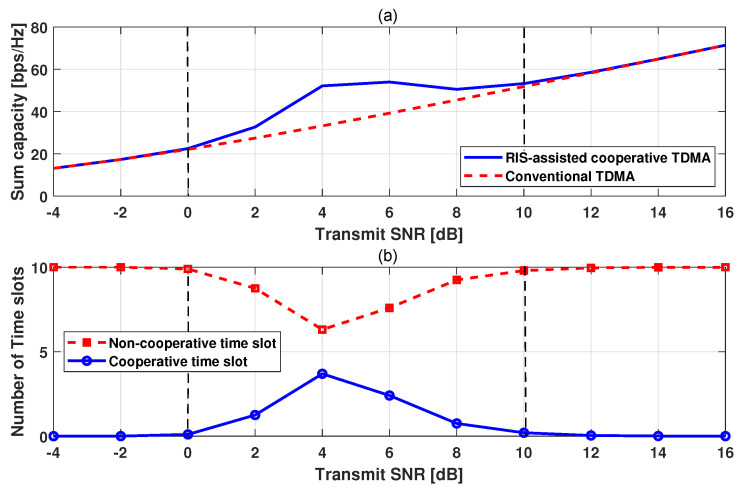
Sum capacity comparison at (**a**) and the numbers of cooperative and non-cooperative time slots at (**b**).

Figure 5 shows the sum capacity of UEs and numbers of cooperative and non-cooperative time slots according to the transmit SNR. As observed in Figure 4, in the moderate SNR region, there exists opportunities for RIS-assisted transmission in cooperative time slots. As shown in Figure 5a, the sum capacity of the RC-TDMA is higher than that of the conventional TDMA in the moderate SNR region. In this region, the number of cooperative times is higher than zero, as shown in Figure 5b. This confirms that the RC-TDMA performs the additional RIS-assisted transmission in the cooperative time slots. In the other SNR region, there are no cooperative time slots. This means that the RC-TDMA operates as the conventional TDMA.

**Figure 6:**
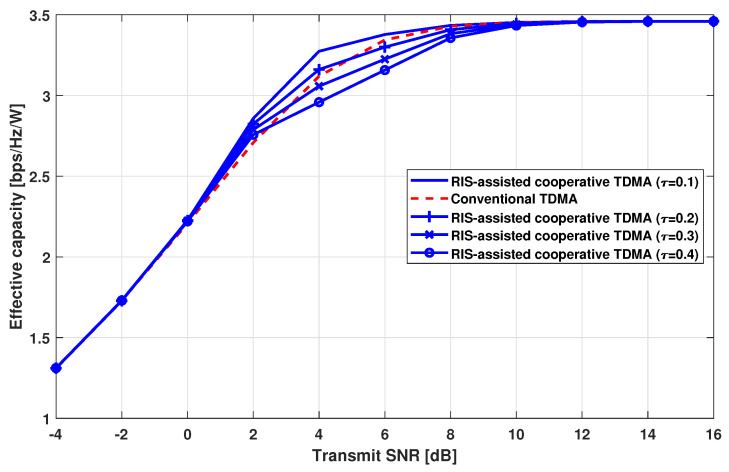
Effective capacity comparison.

Figure 6 shows the effective capacity for comparing power efficiency according to the transmit SNR. Since the RC-TDMA performs the additional RIS-assisted transmission in the moderate SNR region and operates as the conventional TDMA in the other SNR region, the effective capacity shows the same trends as Figure 4 and Figure 5a. In other words, the RC-TDMA shows a higher effective capacity in the moderate SNR when τ=0.1. However, it shows the same effective capacity as the conventional TDMA in the other regions. With increasing τ, the effective capacity of the RC-TDMA decreases in the moderate SNR region. When τ is 0.4, the RC-TDMA shows worse effective capacity than the conventional TDMA. Since τ is the power consumption ratio between BS and the RIS, an increase in τ means that the power consumption at the RIS increases relatively compared with that at BS. Thus, increased power consumption at the RIS leads to inferior power efficiency in the RC-TDMA. However, the proposed scheme can still maintain high efficiency from the perspective of time resources compared with the conventional TDMA.

## 4. Conclusions

This study used a RIS for cooperative transmission to maximize the number of UEs unsatisfied with the QoS in the downlink TDMA system. The simulation and numerical results show that the proposed RC-TDMA guarantees QoS for most UEs compared with a conventional TDMA in a moderate SNR range where UEs that are satisfied with QoS and UEs that are not satisfied are mixed. In addition, from the viewpoint of power efficiency, it was confirmed that effective capacity was high when power consumed in the RIS was lower than that of base stations. Therefore, it was demonstrated that RISs can be fully utilized to ensure QoS without optimally adjusting the phase shift values for passive reflecting elements. 

## Figures and Tables

**Table 1:** RIS-assisted Cooperative TDMA Algorithm.

**Step 1. Initialization**
1: $Count_{1}=Count_{2}=0$, a given random phase-shift diagonal matrix Φ
2: $W_{weak}\left(\in C^{M\times T}\right)=0$, $W_{strong}\left(\in C^{M\times T}\right)=0$, T=ϕ
3: $H_{sort}={\left[h_{1}^{H},\ldots ,h_{K-1}^{H},h_{K}^{H}\right]}^{H}$, where $∥h_{1}∥≦\ldots ≦∥h_{T-1}∥≦∥h_{K}∥$
4: $G_{sort}={\left[g_{1}^{H},\ldots ,g_{K-1}^{H},g_{K}^{H}\right]}^{H}$, where $g_{i}$ is associated with $h_{i}$.
5: **for** *k* = 1: *K* **do**
6: $SINR_{k}=\rho ∥H_{sort}\left(k,:\right){)∥}^{2}$ using (2) and (9)
7: **if** $SINR_{k}<\gamma_{req}$, **then**
8: $Count_{1}=Count_{1}+1$
9: $W_{weak}\left(:,k\right)=\frac{H_{sort}{\left(k,:\right)}^{H}}{∥H_{sort}\left(k,:\right)∥}$ using (9)
10: **else**
11: $Count_{2}=Count_{2}+1$
12: $W_{strong}\left(:,k\right)=\frac{H_{sort}{\left(k,:\right)}^{H}}{∥H_{sort}\left(k,:\right)∥}$ using (9)
13: **end**
14: **end**
15: $N_{c}=\min\left(Count_{1},Count_{2}\right)$
**Step 2. User pairing and Transmit Beamforming Vector Design**
16: **for** *k* = 1: $N_{c}$ **do**
17: Index = 0, TSINR=0
18: **for** *t* = $T-Count_{2}+1$: *T* **do**
19: **if** t∈T **then** go to line 30
20: $w_{c,s}=\max.\;\mathrm{eigenvector}\;\mathrm{of}\;{\left(G_{sort}\left(t,:\right)\Phi H+H_{sort}\left(t,:\right)\right)}^{H}\left(G_{sort}\left(t,:\right)\Phi H+H_{sort}\left(t,:\right)\right)$
using (11)
21: $\rho_{s}=\frac{\gamma_{req}}{{\left(w_{c,s}^{*}\right)}^{H}(G_{sort}\left(t,:\right)\Phi H+H_{sort}{\left(t,:\right)}^{H}\left(G_{sort}\left(t,:\right)\Phi H+H_{sort}\left(t,:\right)\right)w_{c,s}^{*}}$ using (12)
22: $\rho_{w}=\rho -\rho_{s}$ using (13)
23: $w_{c,w}^{max}=\max.\;\mathrm{eigenvector}\;\mathrm{of}\;\left(H^{H}\Phi^{H}G_{sort}{\left(k,:\right)}^{H}G_{sort}\left(k,:\right)\Phi H+H_{sort}{\left(k,:\right)}^{H}H_{sort}\left(k,:\right)\right)$
using (15)
24: $w_{c,w}=\frac{V_{null}V_{null}^{H}w_{c,w}^{max}}{∥V_{null}V_{null}^{H}w_{c,w}^{max}∥}$ using (17), where $V_{null}$ is the null space of $G_{sort}\left(t,:\right)\Phi H+H_{sort}\left(t,:\right)$
25: ${\mathrm{SINR}}_{c,w}=\frac{\rho_{w}{∥\left(G_{sort}\left(k,:\right)\Phi H+H_{sort}\left(k,:\right)\right)w_{c,w}∥}^{2}}{1+\rho_{s}{∥\left(G_{sort}\left(k,:\right)\Phi H+H_{sort}\left(k,:\right)\right)w_{c,s}∥}^{2}}$ using (14)
26: **if** $\mathrm{TSINR}<{\mathrm{SINR}}_{c,w}$, **then**
27: $\mathrm{TSINR}={\mathrm{SINR}}_{c,w}$, $w_{s}=w_{c,s}$ and $w_{w}=w_{c,w}$
28: Index=t
29: **end**
30: **end**
31: $W_{strong}\left(:,Index\right)=w_{s}$ and $W_{weak}\left(:,Index\right)=w_{w}$
32: T=[T,Index]
33: **end**

**Table 2:** Simulation parameters.

Parameter	Value
Number of transmit antennas at BS, *M*	4
Total number of UEs, *K*	10
Total number of time slots, *T*	10
Number of passive reflecting elements, *N*	64
Distance between BS and RIS	500 m
Frequency band	800 MHz
Rician factor	3 dB
Power consumption at BS, $P_{c}$	30 dBm
Power consumption at RIS	$P_{c}\tau ,\;0\leq \tau \leq 1$
Required SINR, $\gamma_{req}$	10 dB

## Data Availability

Data are contained within the article.
